# In Vitro Evaluation of Enteric-Coated HPMC Capsules—Effect of Formulation Factors on Product Performance

**DOI:** 10.3390/pharmaceutics12080696

**Published:** 2020-07-23

**Authors:** Maoqi Fu, Johannes Andreas Blechar, Andreas Sauer, Jozef Al-Gousous, Peter Langguth

**Affiliations:** 1 Department of Biopharmaceutics and Pharmaceutical Technology, Johannes Gutenberg University Mainz, D-55099 Mainz, Germany; maoqifu1@uni-mainz.de (M.F.); jblechar@uni-mainz.de (J.A.B.); joalgous@uni-mainz.de (J.A.-G.); 2 SE Tylose GmbH & Co. KG, D-65203 Wiesbaden, Germany; Andreas.Sauer@setylose.com

**Keywords:** disintegration, biopredictive, enteric-coating, AQOAT, hypromellose acetate succinate (HPMCAS), hypromellose phthalate (HPMCP), Eudragit L100-55, formulation, capsules

## Abstract

A comparative study on different enteric-coated hard capsules was performed. The influence of different formulation factors like choice of enteric polymer, triethyl citrate (TEC) concentration (plasticizer), talc concentrations (anti-tacking agent), and different coating process parameters on the sealing performance of the capsule and the disintegration time were investigated. Furthermore, the influence of different disintegration test methods (with disc vs. without disc and 50 mM U.S. Pharmacopoeia (USP) buffer pH 6.8 vs. biopredictive 15 mM phosphate buffer pH 6.5) was evaluated. All formulations showed sufficient but not equivalent acid resistance when tested. Polymer type was the main factor influencing the capsule sealing and disintegration time. In addition, TEC and talc could affect the performance of the formulation. Regarding the choice of the disintegration test method, the presence of a disc had for the most part only limited influence on the results. The choice of disintegration buffer was found to be important in identifying differences between the formulations.

## 1. Introduction

Acidic polymers are frequently used as enteric coatings in pharmaceutical practice to delay drug release from oral dosage forms, to protect acid labile drugs from the harsh conditions in the stomach or to protect the stomach from irritation by drugs [1,2]. In the acidic gastric media, an enteric polymer is protonated and therefore insoluble. With increasing media pH and bicarbonate molarity along the intestine the polymer gets ionized by deprotonation and becomes water-soluble. Enteric coatings can be applied to different dosage forms, but they are most commonly applied to tablets and capsules.

Enteric-coated (EC) hard capsules are frequently formulated as EC pellets or granules filled into uncoated capsule shells rather than having the filled capsule coated. However, EC capsules with enteric coating of the capsule shell are attractive in early drug development due to the ability to evaluate the performance of drugs that are available only in limited quantities because of the lower formulation effort (e.g., compared to a tablet) [3] or for drugs that are difficult to formulate as a tablet [4,5]. Furthermore, poorly water-soluble drugs or low-dose formulations might be encapsulated as liquid and semi-solid formulations to increase the intestinal absorption or provide a high content uniformity, respectively [6]. In addition, such products are used in cases where the active ingredient is so sensitive that tableting and/or granulation processes could have adverse effects on its stability (e.g., probiotics) [7].

The main challenge in the formulation of enteric-coated hard capsules is the proper sealing of the gap between the body and cap parts of the capsule [8]. There are technologies to seal the capsule with a polymer band [8,9]; however, this is an expensive technology and generally not readily available to manufacturers. The gap part of the capsule can also be closed with a microspray sealing process where a hydroalcoholic solution is applied to the joint between the capsule’s body and cap promoting the fusion of the body and cap parts of the capsule [9]. Another approach to overcome the challenge to properly coat the cap–junction interface is by subcoating the capsules with an immediate release polymer material that helps to seal the gap without having to apply a too-thick enteric coat. This can also help to improve enteric coat adhesion (which is an issue for gelatin rather than hydroxypropyl methylcellulose (HPMC) capsules) [10]. In general, however, a single step enteric coating is more attractive and feasible, particularly for HPMC capsules owing to the good film adhesion on their surfaces [10].

Another technique to achieve gastric resistance was described by Smith et al. who prepared capsules with bulk enteric properties, thus omitting the need of applying an enteric coating [11]. However, in a recent study by our group DRcaps^®^, a commercially available example of capsules with bulk enteric properties [12], was inferior to coated capsules regarding the acid-resistance and showed considerable deformation after 1 h in acidic media [13]. This indicates that it still might be too early for such capsules to obviate the need for enteric coatings.

To date, most research on enteric coatings has been done with tablets or pellets [14,15,16]. Bozdag et al. evaluated the omeprazole release from three enteric formulations, which differ in the polymer used. Furthermore, the weight gain was varied to study the minimum coating thickness needed to achieve a sufficient acid resistance and stability of omeprazole [16]. Nair et al. investigated enteric-coated formulations of esomeprazole tablets. In addition to the variation of the polymer used, the triethyl citrate (TEC) and talc content in the coating were also changed. However, for one polymer only two formulations that differ in the TEC and talc concentration were tested. Consequently, the resulting changes in formulation performance (e.g., drug release) cannot be attributed to one of the additives [14]. Most research focuses on the differences between the polymers used for enteric-coating and only little is known about the influence of additives (e.g., TEC and talc) on the in vitro performance of enteric-coated capsules which present a unique challenge as mentioned before.

The aim of this study is to investigate important formulation factors like concentration of additives and influence of processing parameters on the performance of enteric coatings applied to HPMC capsules. This work thus aims to fill a significant knowledge gap in the selection of the most suitable enteric polymer coupled with appropriate process conditions to result in a product with desired properties.

## 2. Materials and Methods

### 2.1. Materials

Size 0 hydroxypropyl methylcellulose (HPMC) capsules prefilled with maltodextrin (450 mg of maltodextrin per capsule) were received from Lallemand Inc. (Montreal, QC, Canada). Hypromellose acetate succinate (HPMCAS, AS, Shin-Etsu AQOAT^®^) and hypromellose phthalate (HPMCP) were received as gifts from Shin-Etsu (Shin-Etsu Chemical Co. Ltd., Tokyo, Japan). HPMCAS was used as granular low substitution level (-LG), granular medium substitution level (-MG), and granular high substitution level (-HG) (different substitution grades of the polymer) [17] and HPMCP was used as HP-50, HP-55, and HP-55S [18]. Methacrylic acid and ethyl acrylate copolymer (EU 100-55, Eudragit^®^) were used as L100-55 and obtained as free samples from Evonik (Darmstadt, Germany). Cellulose acetate phthalate NF (C-A-P, Aquateric^®^) was received from Eastman via Krahn Chemie GmbH (Hamburg, Germany).Table 1 describes the dissolution pH thresholds and chemical characteristics of the used polymers. Triethyl citrate (TEC) was purchased from Sigma-Aldrich (Bornem, Belgium) and talc (particle size 0.8–35 µm) was obtained from Luzenac SE (Imerys, France). All other chemicals were of analytical grade.

### 2.2. Method

#### 2.2.1. Coating Formulations

The preparation of the coating solution is illustrated by the example of HPMCAS polymer (Shin-Etsu AQOAT^®^). All other polymer solutions were prepared following this method and are described in Table 2, Table 3, Table 4 and Table 5. HPMCAS was dissolved in ethanol (96% *v*/*v*) and stirred for 0.5 h at room temperature. Talc was homogeneously dispersed in approximately 150 mL purified water and afterwards combined with the polymer solution. Thereafter TEC was added to the dispersion. After 1.5 h of ambient stirring the dispersion was filtered through a 200 µm sieve (obtained from VWR International GmbH, Darmstadt, Germany). During the coating experiment the dispersion was gently stirred to prevent sedimentation of talc. Based on dry polymer mass the HPMCAS and HPMCP formulations differed in plasticizer content while the talc content was maintained at a constant level. For the C-A-P formulations the plasticizer content was constant, and the amount of talc varied. The Eudragit batches differed in their processing parameters (spray rate and coating time as well as inlet and outlet processing temperatures).

In formulation E02 a coloring agent (cherry red food dye, Roth GmbH, Bochum, Germany) was used. Due to its low concentration (Table 2) the influence on the mechanical properties and disintegration time is expected to be negligible. Viscosity of coating formulations was determined using a Brookfield LV rotational viscometer (AMETEK Brookfield, Middelboro, MA, USA) at 22–23.5 °C.

The prefilled HPMC capsules were coated in a Solidlab 1 drum coater (Hüttlin GmbH, Schopfheim, Germany) with different polymer formulations. Each batch consisted of 700 mL (0.368 kg) capsules. The process parameters are described in Table 6 and Table 7. The weight gain corresponds to a coating deposition of the dry coating formulation of 10 mg cm^−2^.

#### 2.2.2. Disintegration Test

Enteric-coated capsules were tested in a U.S. Pharmacopoeia (USP) disintegration tester (DT2, SOTAX AG, Aesh, Switzerland) according to the USP chapter <701>. Disintegration test was performed first in an acidic medium for 1 h followed by a buffer medium with near neutral pH at 37 ± 0.5 °C. The volume of the disintegration media was 700 mL and 0.1 N hydrochloric acid pH 1 was used as acidic medium. For the buffer media, simulated intestinal fluid (SIFsp) pH 6.8 (USP buffer) as well as a 15 mM phosphate buffer pH 6.5 (Al-Gousous et al. buffer), that previously demonstrated biopredictivity for enteric-coated tablets [19], were used. The disintegration times recorded are the times at which the capsules ruptured, which helps reduce the uncertainty associated with determining the disintegration times based on “complete disintegration” [20]. Disintegration tests were performed with disks unless otherwise specified. In a previous study a correlation between the disintegration time and dissolution of enteric-coated capsules was found [13]. Therefore, only disintegration tests were performed as surrogate for the drug release from coated capsules.

#### 2.2.3. Acid Uptake Test

Six capsules of each batch were accurately weighed (*W_0_*) and exposed to 0.1 N HCl or 0.01 N HCl (pH 1 and pH 2, respectively) for 1 or 2 h at 37 °C in a disintegration apparatus (see above). Thereafter, the capsules were removed and blotted dry to remove excess liquid on the capsule surface and reweighed (*W_t_*). The percent acid uptake was calculated from the capsule weight difference before and after the test according to Equation (1).

(1) $$\%\;\mathrm{acid}\;\mathrm{uptake}=\frac{W_{t}-W_{0}}{W_{0}}\times 100\;\%$$

#### 2.2.4. Scanning Electron Microscopy (SEM)

SEM analysis was performed on a JEOL JSM-IT100 scanning electron microscope (JEOL Ltd., Tokyo, Japan) equipped with a secondary electron detector in high vacuum mode. The analysis was performed on the body-cap-joint part of the capsules, as well as on the flat face of the cap part using an acceleration voltage of 1 kV and probe current of 30% without prior modification of the sample (e.g., sputtering).

#### 2.2.5. Statistical Analysis

Comparison of different formulation effects on the disintegration time was done using two-way ANOVA for independent samples using the VassarStat website [21]. The first factor (rows) was the used buffer and the second factor (columns) was the tested formulation. The acid uptake of the different formulations was compared by a MANOVA using IBM^®^ SPSS^®^ statistics version 23 (IBM Corporation, Armonk, NY, USA) with the formulation and pH as independent factors, and acid uptake values at 1 and 2 h as the two tested response variables. *p*-values < 0.05 were considered as significant.

#### 2.2.6. Mechanical Properties of the Capsules

The mechanical properties of capsules coated with HPMCAS-MG were tested using a TA.XTplus Texture Analyzer (Stable Micro Systems, Godalming, UK) equipped with a HDP/90 Heavy Duty Platform table (flat insert with target on one side) and 1 cm P/1 KS Kobe stainless steel punch. Basic principles of the test including data analysis and interpretation were published previously [22]. Briefly, the force (N) and the deflection (mm, degree to which the contact point of the capsule shell is displaced under the punch load) were recorded during the test procedure. The data were recorded by the Exponent software (Stable Micro Systems, Godalming, UK). The force exerted and deflection achieved at the fracture point were determined. The area under the stress–strain curve was calculated to compare the energy expended by the process of breaking the film coating. Six capsules from each lot were tested.

## 3. Results and Discussion

### 3.1. Surface Structure of the Enteric-Coated Capsules

**Figure 1:**
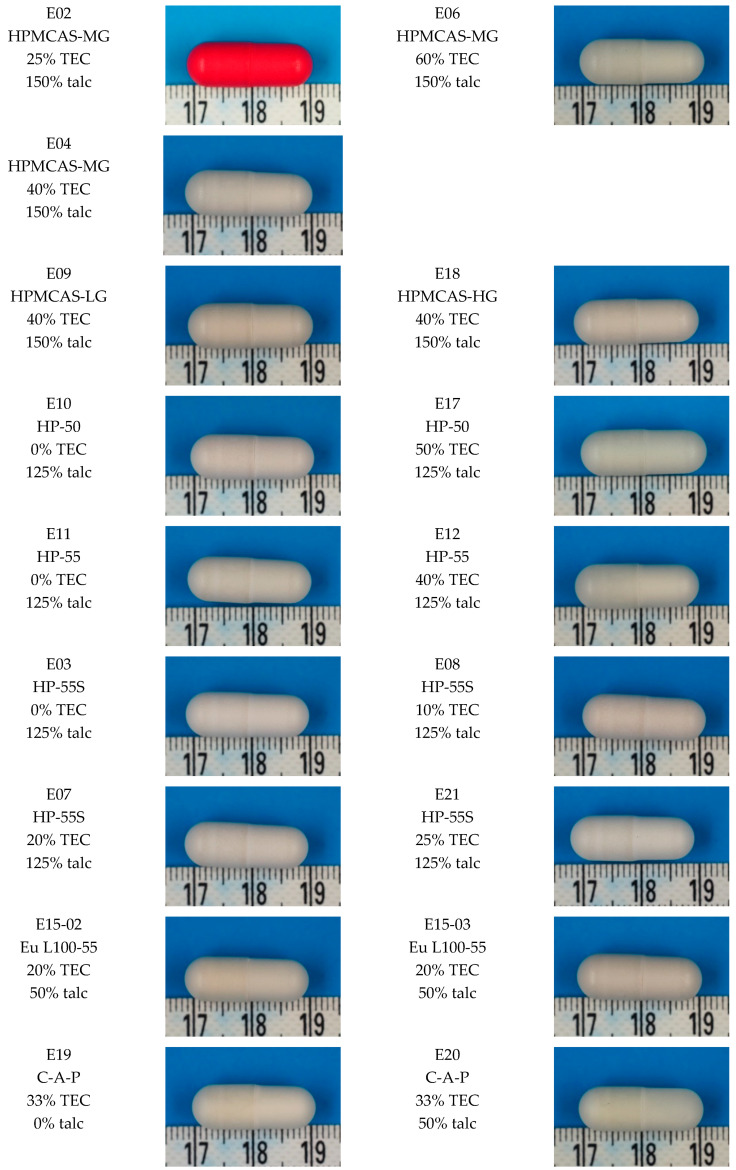
Surface structure of enteric-coated HPMC capsules. % TEC and talc are based on dry polymer weight.

**Figure 2:**
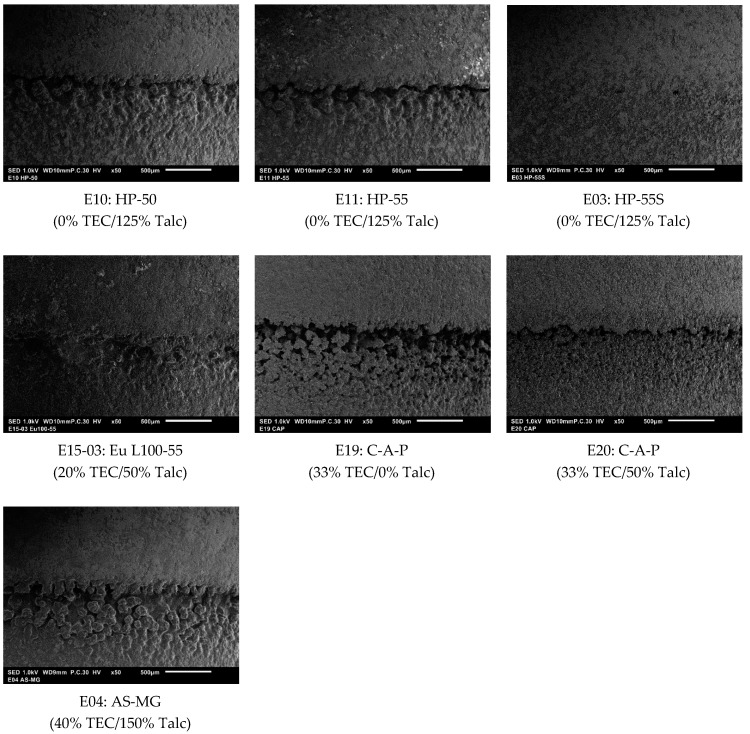
Scanning electron microscopy (SEM) images displaying the gap junction between capsule top and bottom after coating. % TEC and talc are based on dry polymer weight.

All formulations were applied successfully on the capsules and formed a film coating (see Figure 1), although there are visible differences between the different formulations. Within the capsules coated with AS-MG (E02, E04, and E06) and HP-50 (E10 and E17) an increasing amount of TEC resulted in a smoother and brighter surface (Figure 1). This difference was not observed with the HP-55 (E11 and E12) and the HP-55S (E03, E07 and E08) coating (Figure 1). Among the HPMCP series, HP-55S shows the smoothest structure of the coating and the best sealing of the junction between the cap and body of the capsule (Figure 1). This is most probably related to its higher degree of polymerization (reflected by higher viscosity) resulting in greater entanglement and accordingly superior film formation. Actually, HP-55S resulted in the best sealing of the gap among all the studied polymers (see Figure 1 and Figure 2). Between the L100-55 formulations (E15-02 and E15-03) there were no differences observed (Figure 1). Within the C-A-P coatings, talc seems to have a beneficial effect on the surface structure. The coating structure seems smoother in Figure 1 and with higher talc concentration the gap between the body and cap of the capsule is closed. This could be related to the bulking effect of the added talc.

More detailed SEM images were prepared to evaluate the gap closing performance of selected formulations with different polymers (Figure 2). Consistent with the results above, the HP-55S (E03) formulation shows the best sealing of the gap (Figure 2). The film in formulation E03 is smooth and the gap is completely closed. In formulation E10 and E11 the gap is clearly visible and could cause problems in the acid resistance (Figure 2). Coatings of the formulation E15-03 and E04 did close the gap properly, although it is still discernable. Considering the C-A-P formulations (E19 and E20), talc seems to impart a positive effect on the gap closing (Figure 2). In formulation E20 the gap is almost completely closed.

**Figure 3:**
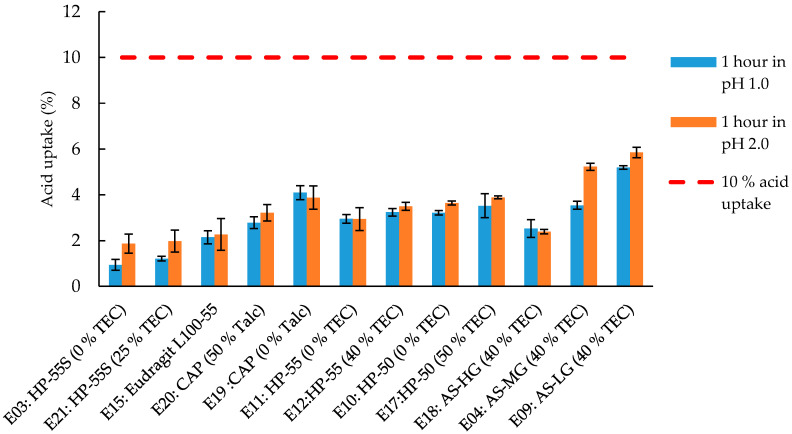
Acid uptake of enteric coated HPMC capsules following incubation in 0.1 N HCl or 0.01 N HCl. Horizontal line represents 10% acid uptake. Given are average acid uptake (%) ± SD; *n* = 6. % TEC and % talc are based on dry polymer weight.

These differences in the completeness of the gap closing can influence the acid resistance of the coated capsules (see results in Section 3.2 and Figure 3). For example, the HP-55S and Eudragit formulations, which show the best sealing of the gap also exhibit the lowest acid uptake values (see Figure 3).

### 3.2. Disintegration Test and Acid Uptake

First, uncoated capsules were tested in 0.1 M HCl. The disintegration time of 5.54 ± 0.3 min demonstrates the need of an enteric coating to provide proper gastric resistance.

After one and two hours of disintegration testing in acidic media, all capsules remained intact with no sign of coat rupture. An effective enteric coating results in low values of acid uptake and values up to 10% acid uptake have been shown to be acceptable for protecting highly acid-labile drugs such as proton pump inhibitors (PPIs) [23]. As shown in Figure 3, all tested formulations showed a sufficient acid resistance at both pH 1 and 2 with an uptake of <10% over one hour of testing, although there were some significant differences (*p*-value < 0.05). HPMCP HP-55S showed the lowest liquid uptake as would be expected from the images in Figure 2.

The two formulations E03 and E21 did not differ significantly after one hour (*p*-value 0.955) but had significant lower acid uptake than all other formulations tested (Figure 3, *p*-value < 0.01). Good results were also obtained with the Eu L100-55 and HMPCAS AS-HG (E18) formulations. Both formulations showed similar acid uptake (*p*-value = 0.754) but differed significantly from all other formulations (*p*-value < 0.05), and only the HP 55S-based formulations showed a better performance. These results are in good agreement with the observations from Section 3.1, where formulation E15 sealed the gap between the capsules body and cap fairly well (Figure 2). However, with decreasing substitution grade of acetyl (and increasing in succinoyl substitution) in the HPMCAS polymer the acid resistance declines. The rank order for acid uptake of the HPMCAS formulations tested is AS-HG < AS-MG < AS-LG with significant difference between the formulations (Figure 3, *p*-value < 0.01). A reason for the decreased acid uptake of the AS-HG could be the increased lipophilicity of the polymer that comes along with the increase in acetyl groups and reduction of succinoyl groups. For the HPMCP formulations E03 and E11 the acid uptake seems to be related to the molecular weight, with the longer chains of HP-55S resulting in a higher degree of entanglement and more coherent film formation enabling better gap sealing (in addition to potentially lower film permeability) compared to the other HPMCP polymers (Figure 3, *p*-value < 0.01). Moreover, the positive influence of talc on the capsule sealing in the C-A-P formulations is reflected in the acid uptake results. Formulation E20 with 50% talc shows a significant lower acid uptake compared to formulation E19 without talc (Figure 3, *p*-value < 0.01).

While gap sealing seems to be an important parameter affecting the acid uptake, it is not the sole factor. For instance, formulation E04 (based on HPMCAS-MG) shows a higher acid uptake compared to formulations E10 and E11 (based on HP-50 and HP-55) even though the SEM did not reveal tangible differences in the degree of gap closing. This most probably has to do with the hydrophobicity imparted by the phthalate groups in HPMCP and its effect on coat swelling and permeability. Yet the degree of gap closure seems to be at least one of the critical parameters influencing the degree to which the coat capsule is acid-proof. This is further illustrated by comparing the surfaces of the two C-A-P-based formulations. The surface of formulation E20 seems to be rougher than that of formulation E19 and exhibits some tiny pores (Figure S1). Nevertheless, formulation E19 exhibits higher acid uptake values (Figure 3) than formulation E20, which demonstrates the importance of the degree to which the gap is sealed.

As expected, the increase in acid uptake after two hours mirrored the trends exhibited during the one hour acid treatment (Figure S2). Comparing the results at pH 1 and pH 2 all capsules did not show significantly different values.

**Figure 4:**
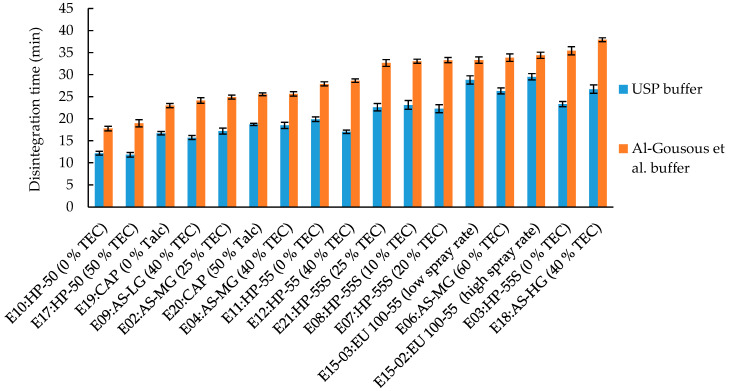
Disintegration times of enteric-coated capsules in 50 mM USP phosphate buffer pH 6.8 and 15 mM phosphate buffer pH 6.5 (Al-Gousous et al. buffer) without disc. Given are average disintegration times ± SD; *n* = 6. % TEC and talc are based on dry polymer weight.

The disintegration times in phosphate-buffered media are depicted in Figure 4. All capsules showed a faster disintegration in the USP media due to the higher capacity of the buffer [10,19,24]. The capsules coated with HP-50 (E10 and E17) exhibit the fastest disintegration in both media, as expected. Among the tested polymers, HP-50 has the lowest dissolution pH threshold (pH >5.0, Table 1) and a lower dissolution pH is generally associated with faster polymer dissolution, although other factors like molecular weight and rheology of the gel layer during dissolution influence the dissolution rate as well. For capsules coated with Eu L100-55, HP-55S, and HPMCAS AS-HG the longest disintegration times were observed.

### 3.3. Comparison of Formulation-Specific Factors on Disintegration Time

Formulation-specific factors at different levels were grouped and compared by statistical analysis, in order to distinguish between significant versus non-significant observations with respect to the effect of coating composition on enteric-coated capsule disintegration times. Analysis was performed using two-way ANOVA.

#### 3.3.1. Effect of Different Polymer Grades on Disintegration Time

The effect of the substitution pattern of HPMCAS and HPMCP was investigated on three formulations for each polymer that just differ in their polymer grade (E09, E04 and E18; and E10, E11 and E03).

**Figure 5:**
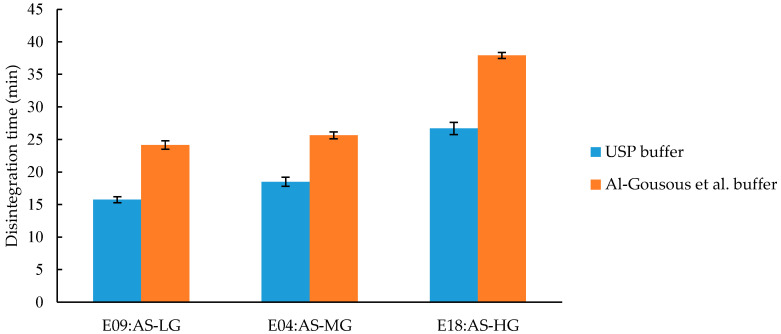
Disintegration time of capsules coated with HPMCAS of different grades (LG, MG, and HG). Given are average disintegration times ± SD; *n* = 6.

Figure 5 shows the disintegration time of the HPMCAS capsules. The observed disintegration times are significantly different (*p*-values < 0.01) between all grades of HPMCAS. In both buffers the rank order of the disintegration times is similar: AS-LG < AS-MG < AS-HG. It is noteworthy, that there is only little difference between the LG and MG disintegration times in both buffers. The polymers AS-LG, AS-MG, and AS-HG have dissolution pH of 5.5, 6.0, and 6.5, respectively [25]. The varying amounts of acetyl (AS-LG < AS-MG < AS-HG) and succinoyl (AS-HG < AS-MG < AS-LG) groups are responsible for different dissolution pH thresholds. An increase in acetyl groups and reduction in succinoyl groups increase the hydrophobicity of the polymer. As a consequence, the polymer requires ionization to a higher degree to become soluble, resulting in a higher opening pH value [26]. Figure S3 shows the acetyl and succinoyl distribution between the polymers. The AS-L and AS-M grade have very similar contents whereas the AS-H grade shows a larger difference to both other polymers which is in line with the experimental disintegration data.

That the solubility and disintegration behavior are not exactly mirror images of each other is not surprising, for the former is a thermodynamic parameter while the latter is a function of a kinetic phenomenon (polymer dissolution). In addition, the dissolution of a polymer (which is a prerequisite for disintegration) is not solely dependent on the polymer solubility behavior but also on other polymer properties like the diffusional and rheological behavior of polymer chains.

**Figure 6:**
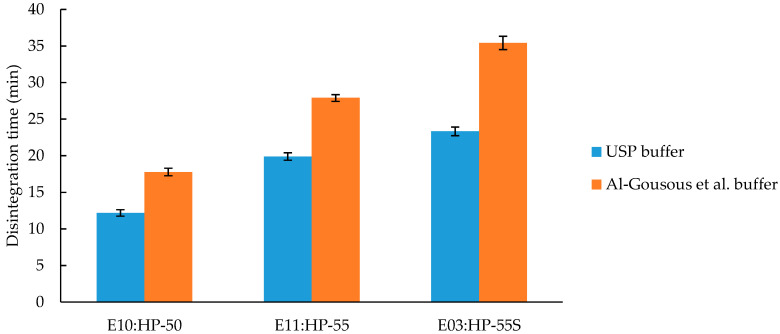
Disintegration time of capsules coated with HPMCP of different grades (HP-50, HP-55, and HP-55S). Given are average disintegration times ± SD; *n* = 6.

HPMCP was tested in three different grades: HP-50, HP-55, and HP-55S. HP 50 has a phthalyl content of 24% and a dissolution pH of 5.0, whereas HP-55 and HP-55S have a phthalyl content of 31% and a dissolution pH of 5.5 [18]. The relationship between phthalyl content and dissolution pH threshold is inverse to the acetyl/succinoyl content in HPMCAS. With more groups that are ionizable the dissolution pH is increased. A possible explanation lies in the bulky hydrophobic nature of the introduced aromatic ring overcoming the solubilizing effect of additional ionizable groups. Referring to the dissolution pH, as expected, the HP-50 disintegration time is lower compared to the HP-55/HP-55S disintegration times (Figure 6). It must be emphasized that the difference in disintegration times of HP-55 and HP-55S was smaller when tested in the USP buffer while the difference is obvious in the more biorelevant Al-Gousous et al. buffer, although all differences between the formulations were significant (*p*-value < 0.01). The difference in disintegration time between HP-55 and HP-55S is due to the molecular weight (resulting in different viscosities, Table 7) of these two polymers. Polymers of the same chemical structure but with higher molecular weight tend to dissolve slower because of greater entanglement and higher gel viscosity [27].

#### 3.3.2. Effect of Plasticizer Content on Disintegration Time

**Figure 7:**
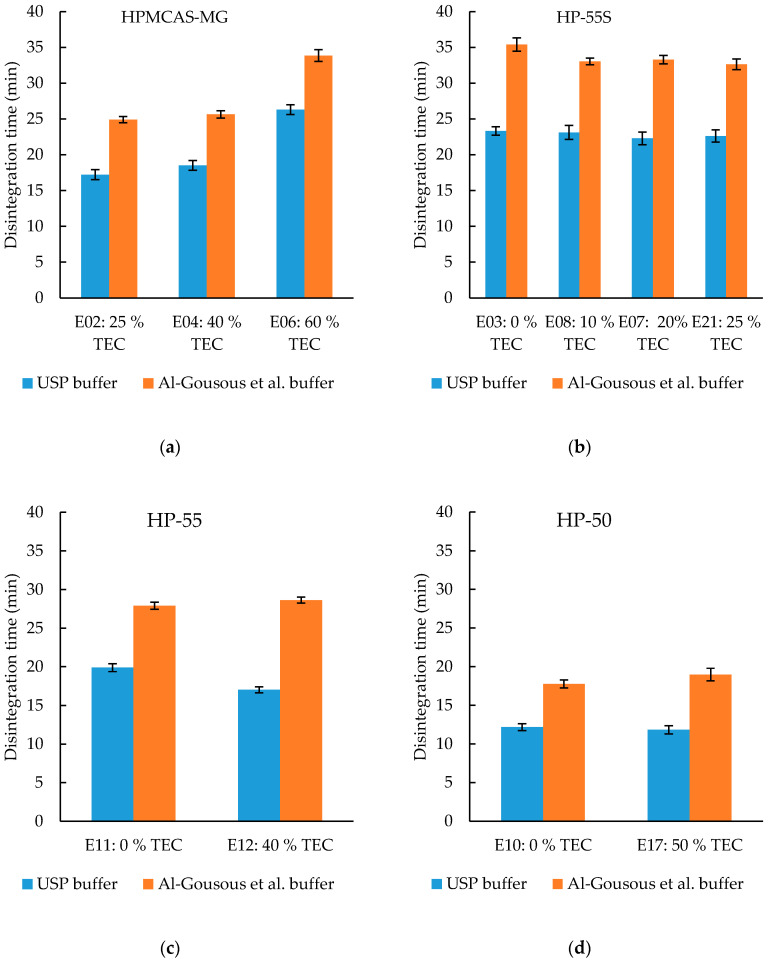
Effect of plasticizer content on the disintegration time in different formulations: (**a**) HPMCAS-MG formulations; (**b**) HP-55S formulations; (**c**) HP-55 formulations; (**d**) HP-50 formulations. Given are average disintegration times ± SD; *n* = 6. TEC content is based on dry polymer weight.

TEC is often used as a plasticizer in pharmaceutical coatings due to its generally recognized as safe (GRAS) status [28]. TEC is a hydrophilic plasticizer which often increases the dissolution of coated formulations. This is in line with the disintegration times of the HP-55S formulations (Figure 7b) in the 15 mM phosphate buffer; although there seems to be a limit for the acceleration of dissolution. The difference in disintegration time in the 15 mM phosphate buffer between 0% TEC and 10% TEC is significant (*p*-value < 0.01), while the difference between 10% TEC and 20% TEC is not (*p*-value = 0.794). Unexpectedly, the disintegration time increased with increasing TEC content in the HPMCAS-MG formulations (Figure 7a). This was observed in both buffers tested. As for the HP-55S polymer, there is a limit above which the disintegration time is not affected by the plasticizer concentration anymore (a possible explanation is outlined in Section 3.3.5). The disintegration times of the HP-55 and HP-50 capsules (Figure 7c,d) do not change with the tested TEC concentrations.

Due to its higher molarity, the USP buffer is less discriminative and could not point out the TEC influence on disintegration for the HP-55S formulation which showcases the importance of using biopredictive buffers. The ANOVA testing with post hoc analysis showed significant differences in the disintegration times of the HP-55S formulations in the 15 mM phosphate buffer, where the higher TEC content led to a slightly faster disintegration most probably because of the pore forming effect of the water-soluble TEC (as well as its disruption of the hydrogen bonds cross-linking the carboxyl groups of the polymer) [29], while this trend was not detected in the USP buffer.

#### 3.3.3. Effect of Talc-Content on C-A-P Formulations on Disintegration Time

**Figure 8:**
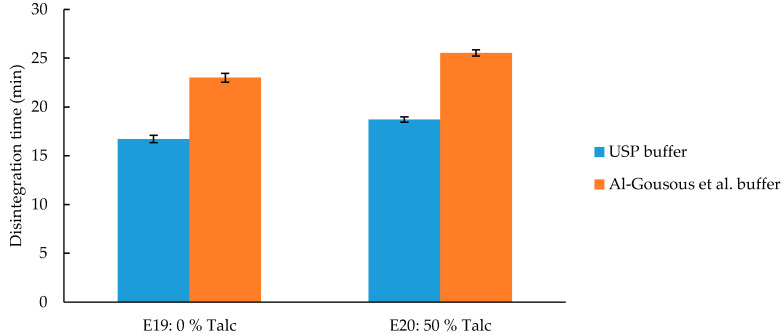
Disintegration time of C-A-P formulations with different talc content. Given are average disintegration times ± SD; *n* = 6. Talc content is based on dry polymer weight.

In pharmaceutical coatings, talc is used as an anti-tacking agent. Due to its lipophilicity high amounts of talc can theoretically delay the disintegration and dissolution of solid oral dosage forms. On the other hand, talc can have a positive effect on sealing the gap of coated hard capsules (see E19 and E20 in Figure 1 and Figure 2). The tested formulations showed only meager, though statistically significant (*p*-value < 0.01), influence of varying talc levels (Figure 8). The hydrophobicity of talc seems to exert a rather weak effect on disintegration time most probably because of the talc particles being coated by polymer chains which reduce their negative effect on wettability.

#### 3.3.4. Effect of Different Processing Parameters on the Disintegration Time of Eudragit L100-55 Formulations

**Figure 9:**
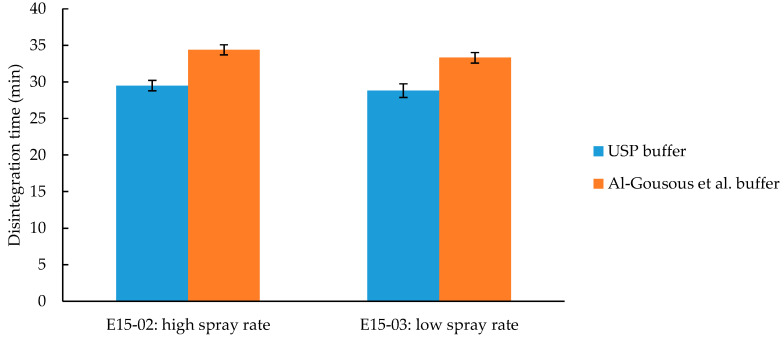
Effect of different coating parameters on the disintegration time. Given are average disintegration times ± SD; *n* = 6. Effect of disc vs. sinker on the disintegration time.

The parameters of the coating process can have various effects on the film formation and therefore on the disintegration and dissolution of a coated dosage form [30]. The influence of two spray rates at different process temperatures on the disintegration time was evaluated. As can be seen in Figure 9, the disintegration times of the two batches are similar (difference approx. 1 min, *p*-value > 0.05). Since the inlet air temperature of both batches was above the minimum film forming temperature (MFT) of 30–35 °C [31], homogeneous films were formed. As long as the coating thickness is comparable and there is no spray drying observed, the disintegration time should not be affected by the different spray rates provided that the process temperature stays above the MFT.

#### 3.3.5. Difference between Disintegration Test Performed with Disc vs. without Disc but with Sinker

**Figure 10:**
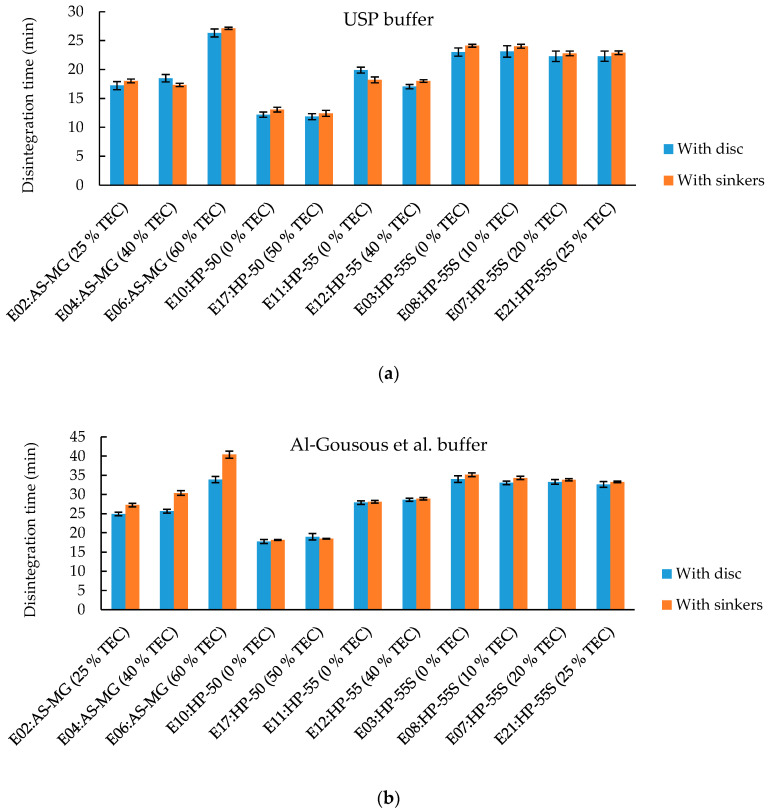
Comparison of disintegration test with disc vs. without disc but with sinker. Given are average disintegration times ± SD; *n* = 6. TEC content is based on dry polymer weight. (**a**) Shows the disintegration times in the USP disintegration media (**b**) Shows the disintegration times in the more biopredictive Al-Gousous et al. buffer.

In disintegration testing a disc is often added to the setup to prevent the dosage form from floating and may introduce mechanical stress on the dosage forms. To investigate whether the mechanical stress on the capsules influences the disintegration times, further disintegration tests were performed without disc but with sinker. The resulting disintegration times are depicted in Figure 10. In the USP buffer only small differences between the disintegration time of the single formulations were observed. Only the HPMCAS-MG capsules consistently showed considerable differences in disintegration time particularly in the Al-Gousous et al. buffer. With increasing concentrations of plasticizer, these differences became more pronounced.

**Figure 11:**
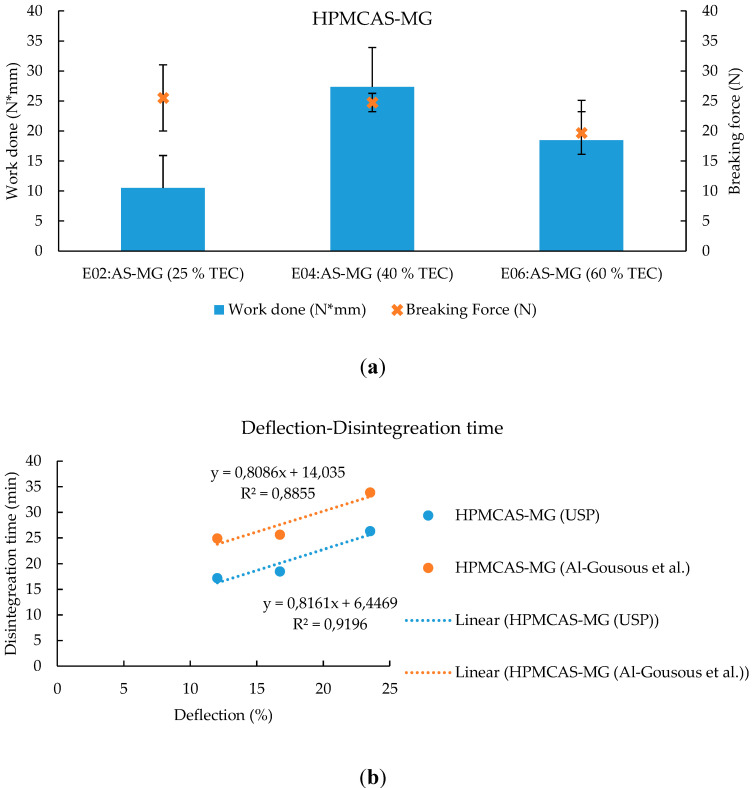
(**a**) Mechanical properties of capsules coated with HPMCAS-MG. Given are the average work done (N*mm) +SD and the breaking force (N) ± SD; *n* = 6. TEC content is based on dry polymer weight. (**b**) Correlation between disintegration time and deflection (% deflection calculated through dividing the observed deflection by the capsule diameter of 7.65 mm).

This provides a potential insight toward explaining the disintegration times increasing with % TEC for the HPMCAS-MG-based formulations. The effect of the presence vs. the absence of disks indicates that mechanical properties can present a significant factor affecting the said disintegration times. When correlating that to the mechanical properties measured by a Texture Analyzer, good correlations between disintegration times and the deflection at break were obtained (Figure 11b). Therefore, it might be presumed that the enhanced film flexibility imparted by increased TEC content had a retarding effect on the disintegration time that outweighed the accelerating effect of the pore formation by the leaching of the water-soluble TEC. No type of correlation could be obtained for the breaking force and energy values. This might have to do with the fact that texture analysis was performed with the capsules being in a dry state, whereas they are soaked with water during the disintegration testing. The resulting confounding effects brought about by the plasticizing effects of water and the leaching of the water-soluble TEC impair the degree to which the measured mechanical properties correlate with the disintegration behavior.

## 4. Conclusions

The performance of various enteric-coating formulations applied to HPMC capsules was investigated, and different performance parameters were evaluated. Regarding the gap sealing, HPMCP HP-55S showed the best performance (which seems to have been reflected in it achieving the best resistance to acid uptake). As for the disintegration times, they varied not only as a function of the polymer used but also as a function of coat formulation additives as well, with anti-tacking agents (talc) and plasticizers (TEC) exhibiting significant degrees of influence. Talc showed a positive effect on the gap closing. TEC can influence the surface smoothness of the coating, the sealing of the junction between the capsules body and cap, and the disintegration time and hence the drug release rate. The influence of TEC on disintegration time can go either in a positive or negative direction, because of the opposing effects of its water solubility on one hand and the enhanced film flexibility that imparts disintegration on the other. Moreover, emphasis should be put on the use of biopredictive methods to detect the influence of formulation factors (e.g., plasticizers) on product performance, where the non-biorelevant compendial medium missed such effects. All in all, film coating of two-piece HPMC capsules has been found to generally be a viable approach toward achieving enteric release properties despite the presence of a gap between the two capsule parts.

## Figures and Tables

**Table 1:** Polymers used in the present study.

Polymer	Name	Grade	Function Related Characteristic	Opening pH Value
Hypromellose acetate succinate, HPMCAS	Shin-Etsu AQOAT^®^	AS-LG	Acetyl: 8.2% Succinoyl: 14.9%	>5.5
Hypromellose acetate succinate, HPMCAS	Shin-Etsu AQOAT^®^	AS-MG	Acetyl: 9.3% Succinoyl: 11.3%	>6.0
Hypromellose acetate succinate, HPMCAS	Shin-Etsu AQOAT^®^	AS-HG	Acetyl: 11.7% Succinoyl: 7.5%	>6.5
Hypromellose Phthalate	HPMCP	HP-50	Phthalyl: 23.1% Viscosity: 55 mPas	>5.0
Hypromellose Phthalate	HPMCP	HP-55	Phthalyl: 32.9% Viscosity: 43 mPas	>5.5
Hypromellose Phthalate	HPMCP	HP-55S	Phthalyl: 33.2% Viscosity: 167 mPas	>5.5
Methacrylic acid and ethyl acrylate copolymer	Eudragit^®^	L100-55	Ratio of methacylic acid to ethyl acrylate ~1:1	>5.5
Cellulose acetate phthalate	Eastman^TM^ C-A-P	C-A-P Cellulose Ester NF	Acetyl: 21.5–26% Phthalyl: 30–36%	>6.0

**Table 2:** Composition of HPMCAS coating dispersion. All concentrations are given in percent (*w*/*w*).

	HPMCAS				
Batch No.	E02	E06	E04	E09	E18
Polymer type	AS-MG	AS-MG	AS-MG	AS-LG	AS-HG
Content	4.99	5.00	5.00	5.00	5.00
Triethyl citrate (TEC)	1.25	3.00	2.00	2.00	2.00
Talc	7.49	7.50	7.50	7.50	7.50
Water	17.23	16.90	16.50	16.50	16.50
Ethanol 96% (*v*/*v*)	68.95	67.60	69.00	69.00	69.00
Food color	0.09	0	0	0	0
Total	100.00	100.00	100.00	100.00	100.00

**Table 3:** Composition of HPMCP coating dispersion. All concentrations are given in percent (*w*/*w*).

	HPMCP							
Batch No.	E03	E08	E07	E21	E11	E12	E10	E17
Polymer type	HP-55S	HP-55S	HP-55S	HP-55S	HP-55	HP-55	HP-50	HP-50
content	6.00	6.00	6.00	6.00	6.00	6.00	6.00	6.00
TEC	0.00	0.60	1.20	1.50	0.00	2.40	0.00	3.00
Talc	7.50	7.50	7.50	7.50	7.50	7.50	7.50	7.50
Water	12.97	17.90	17.00	17.00	12.97	17.90	12.97	16.70
Ethanol 96% (*v*/*v*)	73.53	68.00	68.30	68.00	73.53	68.00	73.53	66.80
Total	100.00	100.00	100.00	100.00	100.00	100.00	100.00	100.00

**Table 4:** Composition of Eudragit^®^ coating dispersion. All concentrations are given in percent (*w*/*w*).

	Methacrylic Acid and Ethyl Acrylate Copolymer	
Batch No.	E15-02	E15-03
Polymer type	EU 100-55	EU 100-55
content	6.00	6.00
TEC	1.20	1.20
Talc	3.00	3.00
Water	14.00	14.00
Ethanol 96% (*v*/*v*)	75.80	75.80
Total	100.00	100.00

**Table 5:** Composition of C-A-P coating dispersion. All concentrations are given in percent (*w*/*w*).

	Cellulose Acetate Phthalate	
Batch No.	E19	E20
Polymer type	C-A-P	C-A-P
content	6.00	6.00
TEC	2.00	2.00
Talc	0.00	3.00
Water	46.00	44.50
Ethanol 96% (*v*/*v*)	46.00	44.50
Total	100.00	100.00

**Table 6:** Operating parameters for coating of HPMC capsules with enteric polymers on the drum coater.

	Shin-Etsu AQOAT^®^/HPMCP	Eudragit^®^ L100-55	Aquateric^®^
Before coating			
Preheating to °C	30	25	30
Coating			
Nozzle diameter (mm)	0.5	0.5	0.5
Spray rate (g/min)	6.5–7	0.75–0.85	7
Atomizing pressure (bar)	2.0	0.5	2.0
Inlet air volume (m^3^/h)	55	60	50
Inlet air temperature (°C)	58–60	40–75	55
Product temperature (°C)	35–38	25–40	37
Coating time (min)	65	181–310	80
Drum speed (rpm)	30	30	30

**Table 7:** Processing parameters for each formulation. The order in the table is given by the type of polymer used, starting with HPMCAS series (E02–E18), followed by HPMCP series (E03–E17) and Eudragit (E15-02–E15-03) and C-A-P (E19–E20). Weight gain is the increase in mass due to applied polymer based on the uncoated filled capsule.

Batch Name	Viscosity (mPas)	Spray Rate (g/min)	Inlet Temp. (°C)	Outlet Temp. (°C)	Weight Gain (%)
E02	32.6 ± 0.1	6.5–7.0	57–64	38–39	7.10
E06	32.6 ± 0.1	6.9–8.0	59–62	39–41	7.20
E04	32.6 ± 0.1	6.5–8.5	58–60	39–40	7.15
E09	29.6 ± 0.1	6.3–7.4	58–60	39–40	7.26
E18	40.2 ± 0.2	8.6–10	57–60	38–40	7.23
E03	99.2 ± 0.2	6.5–7.3	58–60	39–40	6.95
E08	99.2 ± 0.2	7.2–9.0	59–60	39–41	7.21
E07	99.2 ± 0.2	6.6–8.2	59–64	39–42	7.06
E21	99.2 ± 0.2	7.1–7.9	58–60	40–41	7.12
E10	48.1 ± 0.2	6.0–7.1	59–61	39–42	7.17
E11	46.0 ± 0.2	5.3–6.7	59–64	39–40	7.28
E12	47.5 ± 0.2	6.7–8.2	58–62	38–41	7.25
E17	48.8 ± 0.2	7.0–8.9	58–60	39–40	7.09
E15-02	45.5 ± 0.2	2.7–2.8	73–80	45–48	7.40
E15-03	45.2 ± 0.2	1.2–1.3	35–40	24–31	7.50
E19	41.5 ± 0.2	4.4–4.9	55–62	39–40	7.05
E20	47.2 ± 0.2	4.6–5.0	46–52	35–38	7.13

## Supplementary Materials

The following are available online at https://www.mdpi.com/1999-4923/12/8/696/s1, Figure S1: SEM images displaying the surface of the coated capsules. % talc is based on dry polymer weight, Figure S2: Acid uptake of enteric coated HPMC-capsules following incubation in (a) 0.1N HCl or (b) 0.01N HCl. Horizontal line represents 10 % acid uptake. Given are average acid uptake (%) ± SD; n=6. % TEC and % talc are based on dry polymer weight., Figure S3: Acetyl- and succinoyl content of the different HPMCAS-grades.
