# Supplementary material for: In Vitro Evaluation of Enteric-Coated HPMC Capsules—Effect of Formulation Factors on Product Performance
Source: Pharmaceutics. 2020 Jul 23;12(8):696. doi: 10.3390/pharmaceutics12080696 (PMC7465055; doi:10.3390/pharmaceutics12080696)
Supplement: Supplementary file 1 [file pharmaceutics-12-00696-s001.pdf]

pharmaceutics

In Vitro Evaluation of Enteric-Coated HPMC Capsules—Effect of Formulation Factors on Product Performance

Maoqi Fu, Johannes Andreas Blechar, Andreas Sauer , Jozef Al-Gousous and Peter Langguth

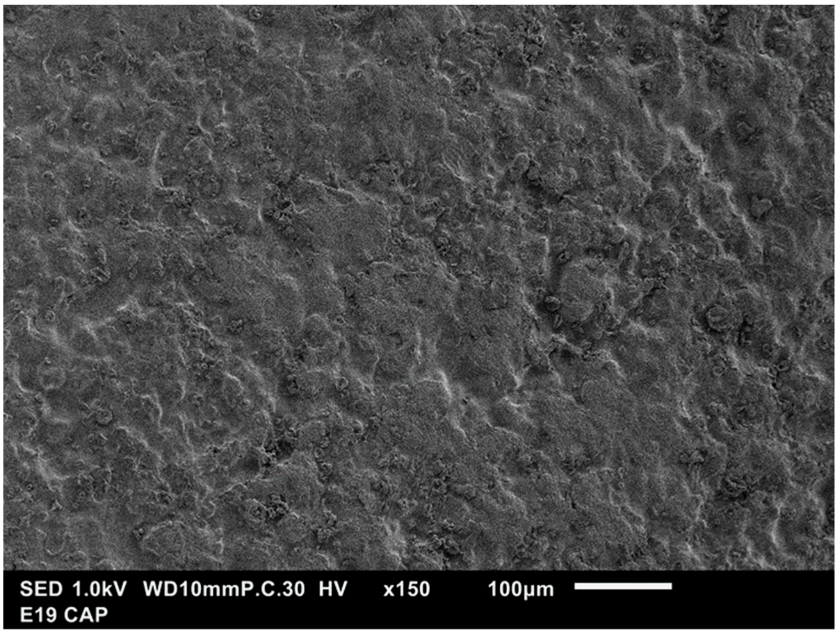

E19: C-A-P (33 % TEC/0 % Talc)

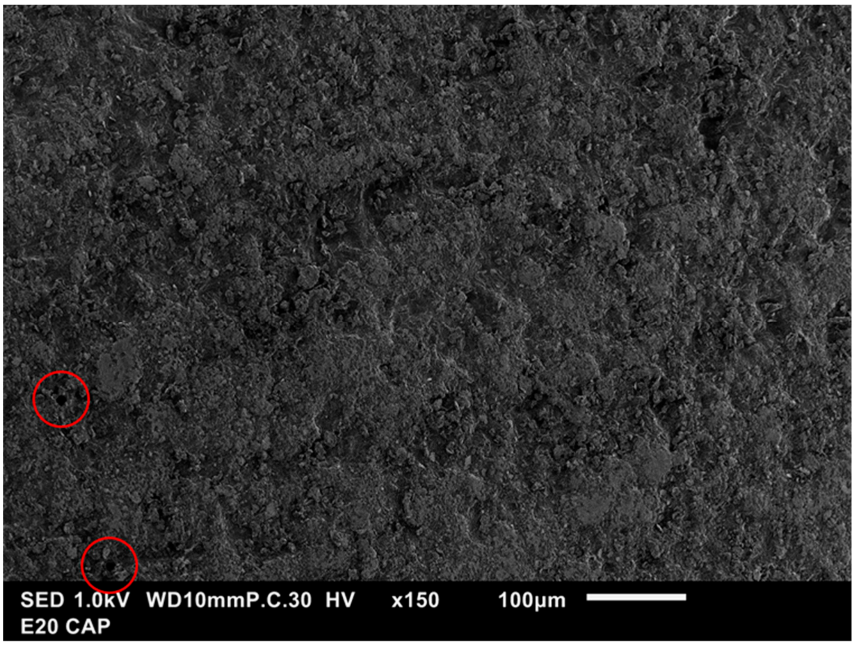

E20: C-A-P (33 % TEC/50 % Talc)

Figure S1. SEM images displaying the surface of the coated capsules. % talc is based on dry polymer weight.

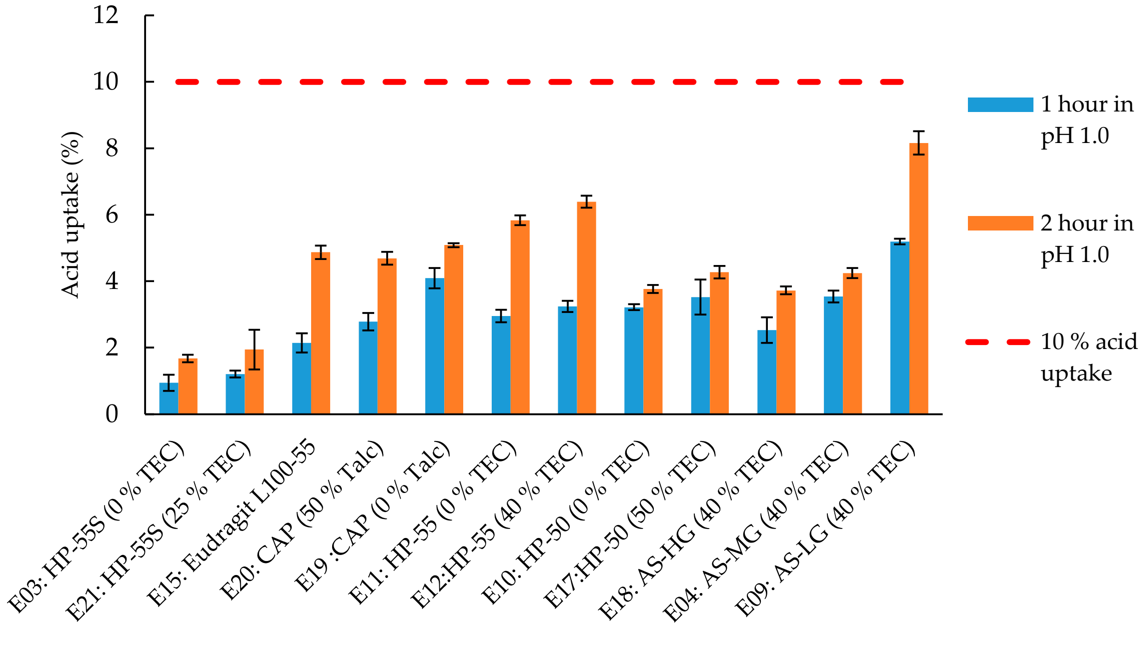

(a)

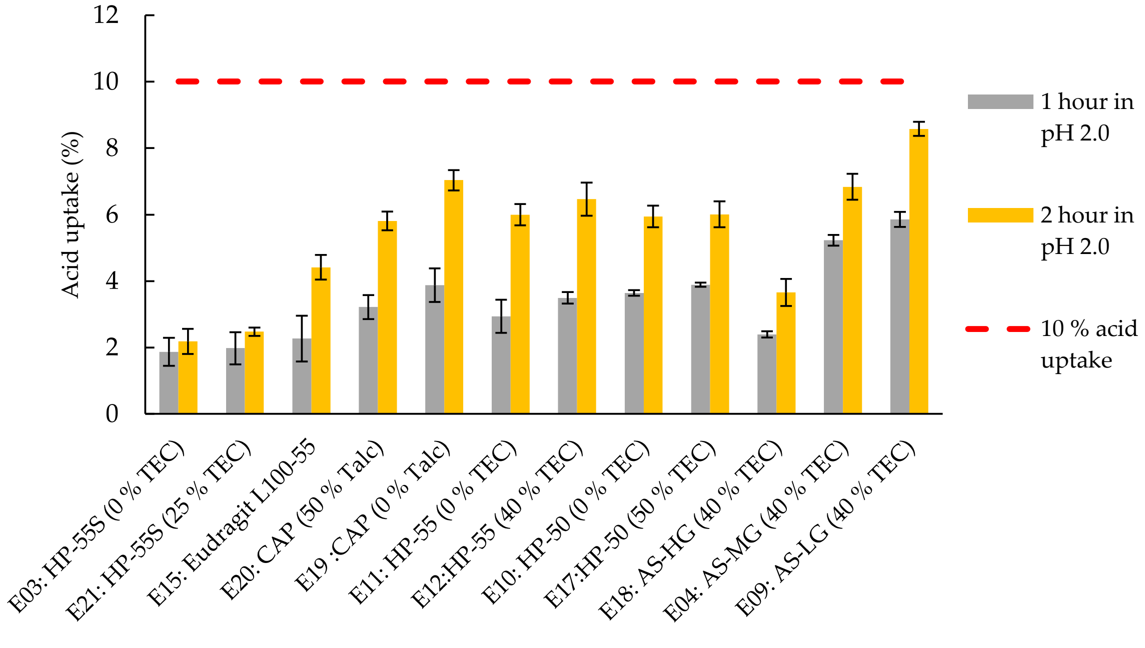

(b)

Figure S2. Acid uptake of enteric coated HPMC-capsules following incubation in 0.1N HCl (a) or 0.01N HCl (b). Horizontal line represents 10 % acid uptake. Given are average acid uptake (%) ± SD; n=6. % TEC and % talc are based on dry polymer weight.

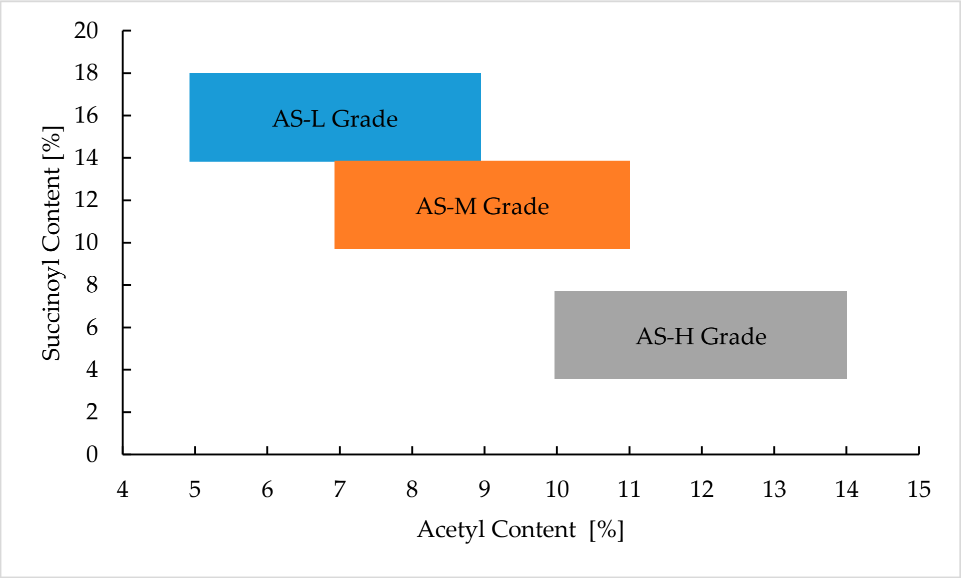

Figure S1. Acetyl- and succinoyl content of the different HPMCAS-grades [1,2].

References:

1. Shin-Etsu Chemical Co. Ltd. Shin-Etsu Pharmaceutical Excipients Available online: https://www.metolose.jp/en/pharmaceutical/ (accessed on 30 March 2020).
2. Ashland Chemical Co. Ltd. AquaSolve hydroxypropylmethylcellulose acetate succinate Available online: https://www.ashland.com/file_source/Ashland/Industries/Pharmaceutical/Links/PC-12624.6_AquaSolve_HPMCAS_Physical_Chemical_Properties.pdf (accessed on 10 April 2020).

© 2020 by the authors. Submitted for possible open access publication under the terms and conditions of the Creative Commons Attribution (CC BY) license (http://creativecommons.org/licenses/by/4.0/).
